# Technological Strategies for the Patient Experience in Emergency Departments: Scoping Review

**DOI:** 10.2196/79782

**Published:** 2026-03-09

**Authors:** Andrés Santiago Santafé, Juan Manuel Aranda, Wilmer Jair Beltrán, William Javier Guerrero, Maryory Guevara Lozano, Ingrid Xiomara Bustos

**Affiliations:** 1 Faculty of Engineering, Universidad de La Sabana, Campus del Puente del Común, Km 7 Autopista Norte de Bogotá, Chía, Cundinamarca, 250001, Colombia, 57 6018615555 ext 25108; 2 Faculty of Nursing and Rehabilitation, Universidad de La Sabana, Chía, Cundinamarca, Colombia; 3 Clínica Universidad de La Sabana, Chía, Cundinamarca, Colombia

**Keywords:** scoping review, PRISMA-ScR framework, emergency department, patient journey, communication technology, patient experience

## Abstract

**Background:**

Technology has improved patient care in hospitals, enhancing the overall patient experience. However, digitalization raises questions on effectively integrating technological strategies to ensure assertive communication of information during emergency department (ED) journeys. Keeping patients well-informed boosts their service perception and satisfaction, a factor often neglected by institutions in EDs.

**Objective:**

This paper analyzes relevant studies on technological strategies designed for EDs aimed at improving patient experience, focusing on communication and information access. We analyze the technologies, outcomes, impacts, and challenges of the strategies.

**Methods:**

A scoping review was conducted using the PRISMA-ScR (Preferred Reporting Items for Systematic Reviews and Meta-Analyses Extension for Scoping Reviews) guidelines and CADIMA tool. Searches were performed in Scopus, PubMed, IEEE Xplore, and CINAHL databases. Articles published from January 2018 to December 2024 were included. Quality appraisal was performed using the Crowe Critical Appraisal Tool version 1.4. Three reviewers independently examined the title and abstract for eligibility based on the inclusion and exclusion criteria.

**Results:**

Sixteen eligible studies were included. Four technological strategy categories were identified: artificial intelligence−based, simulation-based, infrastructure and hardware technologies, and interfaces and information systems. Mobile and web applications were the main technologies adopted in the studies.

**Conclusions:**

Technological strategies hold significant potential to enhance patient experiences in EDs by providing real-time updates on medical status and care progress. However, their effectiveness depends on usability, literacy, and system design. Existing literature highlights the impact and challenges of deploying and using these strategies in EDs. However, no studies have systematically evaluated long-term outcomes or cost-effectiveness across diverse ED settings.

## Introduction

The emergency department (ED) constitutes the gateway to health care for many people, being the place where treatment begins for a wide spectrum of diseases, some of which are life-threatening and require immediate attention [1]. The typical patient journey in the ED involves triage classification, medical evaluation, necessary examinations, medications, or procedures, reevaluation, and finally discharge or observation [2]. However, challenges such as overcrowding, uncomfortable facilities, and poor communication continue to hinder timely and efficient ED patient care, negatively impacting both patient experience and institutional performance [3]. In fact, effective and assertive communication of information about the ED patient journey has been found to be associated with patient satisfaction; thus, studies indicated that the lack of information on the progress and delays has a greater effect on patient satisfaction than perceived waiting times while visiting the ED [4]. In addition, it leads to feelings of powerlessness for patients and their family members [5]. Patients who feel well informed tend to have a better perception of the service [6], leading to higher satisfaction with the care and reduced anxiety [7], which in turn contributes to greater institutional efficiency [8].

With recent technological innovations, the potential to address communication challenges in EDs has increased significantly. Technologies that enhance communication and improve information accessibility have shown promise in improving the patient experience in the ED [9]. However, due to the rapid pace of technological advancement and the growing use of digital solutions in health care, there is an urgent need to review existing evidence to guide future implementation efforts.

This scoping review aims to analyze relevant studies and describe the evidence on technological strategies implemented in EDs worldwide that enhance patients’ experiences during their journey in the ED. A technological strategy refers to the approach and implementation of technologies and supporting infrastructure within ED workflows to improve patient experience throughout their journey. Regarding the ED patient experience, the review focuses on two dimensions: communication and access to information. Communication refers to the exchange of timely, accurate, and comprehensible information between health care providers and patients or family members regarding medical status and progress updates during the ED visit. Access to information refers to patients’ ability to obtain and understand relevant details about their journey in the ED. The following research questions (RQ) were addressed:

- What technological strategies exist to improve patient experience in EDs, considering dimensions such as communication and information accessibility?
- What infrastructure and technologies support technological strategies?

The scoping review offers a comprehensive and interdisciplinary perspective, integrating insights from clinical, technological, and human-centered design domains. The primary innovation of this study lies in its integrative and multidimensional approach to evaluating technological interventions in EDs, specifically through the lens of patient communication and information accessibility—dimensions often underrepresented in prior reviews. Unlike earlier works that focus predominantly on clinical outcomes or operational efficiency, this review foregrounds the patient experience and satisfaction as central evaluative criteria. It also identifies a critical knowledge gap: the lack of standardized frameworks for evaluating the long-term effectiveness, cost-efficiency, and scalability of these technologies across diverse health care settings. Additionally, the review highlights the insufficient attention given to the adaptability of these tools for vulnerable populations, such as those with low digital literacy or disabilities, and the need for participatory design approaches to ensure equitable and sustainable implementation.

## Methods

### Databases and Search Keywords

To analyze the current and future landscape of technological strategies in EDs, a scoping review was conducted following the PRISMA-ScR (Preferred Reporting Items for Systematic Reviews and Meta-Analyses Extension for Scoping Reviews) guidelines [10]. Studies were retrieved from Scopus, PubMed, IEEE Xplore, and CINAHL in January 2025. The inclusion of CINAHL and PubMed ensures coverage of nursing and clinical perspectives that are highly relevant to patient care and workflow improvements, complementing the technical focus of IEEE Xplore and the multidisciplinary scope of Scopus. The keywords used in the search queries included technology, strategy, ED, communication, and access to information. The complete search strategy was as follows: (“Technolog*”) AND (“Strateg*” OR “Approach*” OR “Solution*” OR “Framework*” OR “System*” OR “Model*”) AND (“Emergency Department*” OR “Emergency Service, Hospital” OR “Emergency Room*” OR “Emergency Medical Service*”) AND (“Communication” OR “Access to Information”). The final database search queries for the period January 2018 to December 2024 are summarized in Multimedia Appendix 1.

### Inclusion and Exclusion Criteria

To ensure the quality of the articles, only those that met the inclusion and exclusion criteria presented in Textbox 1 were considered in the review process. These criteria were chosen to focus on studies that specifically examine technological strategies designed for EDs and their impact on patient experience, particularly in the dimensions of communication and information accessibility. Three reviewers independently applied the criteria to refine the list of eligible articles. In cases of uncertainty, the decision was made collectively after discussion.

Textbox 1. **Inclusion and exclusion criteria.**

**Inclusion criteria**

- Documents published between 2018 and 2024 (including both years)
- Documents published in peer-reviewed journals or conference proceedings
- Full-text articles available in English
- The research setting is at least a hospital or an emergency department (ED), regardless of the age or sex of the patients who have used the ED
- Technological strategies specifically designed for EDs aimed at improving the patient’s experience during their ED journey, focusing on communication and access to information
- Studies reporting on at least one patient-centered outcome (satisfaction, experience, or well-being)

**Exclusion criteria**

- Technological strategies outside the health care sector or those presented as telemedicine, remote medicine, or prehospital solutions
- Patients who have not used hospital or ED services
- Articles that do not present a descriptive evaluation, case study, or empirical evidence regarding the design, implementation, or impact of technological interventions on patient experiences in EDs
- Protocol-only studies or incomplete data reports

### Study Selection

A 3-step filtering process was implemented to refine the selection of studies relevant to our RQ:

- In the first filter, *identification*, duplicates were removed. This process ensured the uniqueness of each record for accuracy and to avoid redundancy.
- The second filter involved *screening* by title and abstract, which significantly narrowed down the selection, based on our inclusion and exclusion criteria. This step focuses on identifying papers that are most relevant to ED’s technological advancements.
- The third filter involved a full-text review to assess the *eligibility* of studies that addressed our RQ, culminating in the final selection of studies included in the review.

CADIMA was used for both the first and second filtering stages. CADIMA is an open-access online tool developed by the Julius Kühn-Institut during the EU-funded Global Response Against Child Exploitation project to support the conduct of systematic reviews [11].

### Data Charting

The data charting process for the studies included in this review was structured into three rigorous rounds to explore the categories of technological strategies in EDs comprehensively. In the first round, we extracted fundamental attributes from each study, such as the authors, publication year, journal, and keywords. This process allowed us to understand the distribution of research over time and identify key categories within the field. A standardized data charting form was jointly developed in Microsoft Excel by three reviewers and piloted on a subset of studies to ensure consistency and clarity before full data extraction.

In the second round, we delved deeper into technological strategies applied in the studies. The charting form included variables such as the affected dimension (communication or access to information), technology and infrastructure used, impact on patient experience, measurement tools for usability or satisfaction, preferred information by patients, implementation details (approach, settings, challenges), and implementation outcomes.

Finally, in the third round, each paper was independently reviewed by three reviewers to ensure consistency and reduce bias in the analytical process. Any discrepancies were discussed and resolved by consensus. The charting process was iterative, and adjustments were made to the Excel form when new variables or categories emerged.

### Quality Appraisal

Each selected paper underwent a quality appraisal conducted independently by two reviewers using the Crowe Critical Appraisal Tool version 1.4 (CCAT) [12]. This step was undertaken to assess the methodological rigor and credibility of the included evidence, supporting a more reliable interpretation of the findings in relation to our research questions. The CCAT is a widely used instrument recognized for its high reliability in evaluating research papers [13]. This instrument is organized into 8 categories comprising 22 items. Each category is scored on a 6-point scale ranging from 0 (lowest) to 5 (highest). The overall score is calculated as a percentage, and we arbitrarily considered acceptable a score above 60%. Prior to the appraisal, both reviewers were familiarized with the CCAT manual to ensure consistent interpretation of criteria. In cases of discrepancies between the reviewers’ assessments, consensus was reached through discussion of the arguments and evidence.

### Synthesis of Results

We grouped the studies by technological strategy category and summarized their associated challenges, as well as related patient experience outcomes and impacts. The strategy categories were defined using a content-based grouping approach: we reviewed all included studies, examined the technologies and their stated purposes, and clustered similar technologies based on their primary function and orientation toward improving ED patient experience. The synthesis mapped the relationship between each strategy and its impact on communication and access to information, highlighting key outcomes, impacts, and challenges. Technologies and IT infrastructures were tabulated by strategy category, while patients’ preferred information and satisfaction improvements were synthesized narratively.

## Results

### Flow Diagram and Number of Studies

**Figure 1.**
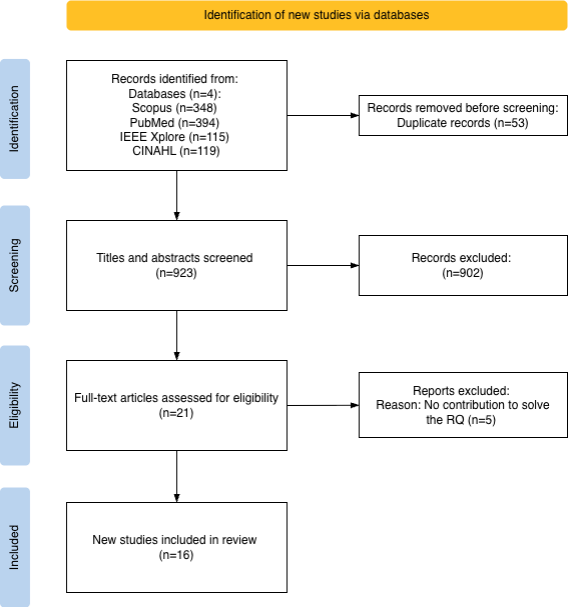
Flow diagram of the scoping review process. RQ: research question.

The scoping review process is detailed in the flow diagram shown in Figure 1, presenting the stepwise filtering and the number of studies included at each step. In the *identification* step, 976 papers were identified and selected. Papers were imported into the CADIMA, where duplicates were removed, resulting in 923 papers. These papers were *screened*, applying the defined inclusion and exclusion criteria, where 21 papers were classified as eligible. Finally, after *full-text* reading, 16 papers were selected for further analysis; 5 articles did not contribute to the RQ.

### General Description and Characteristics of Studies

The set of 16 studies, published between 2018 and 2024 in journals such as *Journal of Medical Internet Research* and *Applied Clinical Informatics*, examines a range of technological strategies designed to improve patient experience in EDs. These studies address key aspects, including the design and implementation of solutions, the technological and infrastructural components that support them, patients’ preferences for information delivery, system usability, and the assessment of their impact on patient satisfaction and overall experience. Methodologically, the articles include qualitative evaluations, observational pilot studies, and mixed methods research, reflecting a diversity of approaches (see Multimedia Appendix 2).

The reviewed literature reveals an interdisciplinary orientation, with collaborations among computer scientists, clinicians, designers, and data analysts. Notably, studies originate from diverse regions, including the United States [14-19], the United Kingdom [20], Italy [21], Israel [22,23], Denmark [5], Australia [24], Thailand [25], Malaysia [26], South Korea [27], and Greece [28]. Finally, the quality appraisal of the 16 studies revealed that 87.5% (n=14) were of high quality and 12.5% (n=2) of medium quality (see Multimedia Appendix 2).

### Key Findings on Technological Strategies

#### Technological Strategies

Based on revised studies and following the process described in the Synthesis of Results subsection, we categorized the technological strategies that help to enhance patient journey experiences in EDs into four primary categories, as outlined below.

1. Interfaces and information systems: This category encompasses tools and information systems designed to provide access to comprehensive patient medical information and ED stage updates, enhancing patients’ understanding of their journeys through the ED and facilitating communication with medical staff.
2. Infrastructure and hardware technologies: This category includes strategies focused on the underlying IT infrastructure necessary for ED operations. It encompasses advanced computing systems, integration devices, and hardware that are critical for patient care and tracking.
3. Artificial intelligence (AI)–based strategy: This category includes strategies that utilize machine learning (ML) and deep learning models for predictive analytics and pattern recognition in EDs.
4. Simulation-based strategy: This category encompasses simulation strategies, which are essential for evaluating the performance of health care operations, analyzing patient flow, and modeling complex interactions within ED settings.

**Figure 2.**
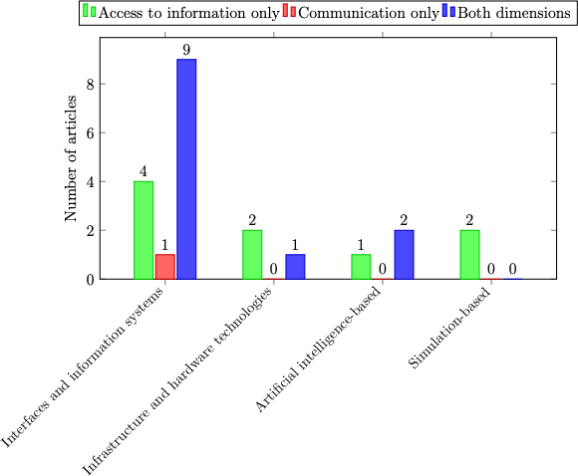
Distribution of articles by technological strategy category and dimension focus.

Figure 2 illustrates the distribution of these strategy categories by dimension within the reviewed literature. Interfaces and information systems strategies were the most frequently cited, appearing in 14 studies, followed by infrastructure and hardware technologies and AI-based strategies, each appearing in 3 studies, and the simulation-based category, appearing in 2 studies. All categories addressed both dimensions, except the simulation-based category. These findings indicate a clear preference for integrated solutions and reveal a gap in studies dedicated exclusively to communication strategies.

Table 1 provides a comprehensive overview of the key technological strategies implemented in EDs to enhance patient experience. For each strategy, the table details the specific dimension(s) addressed (communication and access to information), the implementation approach used and the target population (patients, family members, or health care staff), the measurement methods used to assess outcomes, the observed outcomes and their impact on patient experience and satisfaction, and the implementation challenges encountered. The results reveal a strong emphasis on systems that integrate both communication and access to information, particularly through interfaces and information systems such as digital whiteboards [16], clinical logic platforms [5], and discharge tools [17,24]. These solutions consistently aim to enhance patient and family engagement, improve satisfaction, and streamline information flow during care transitions [22].

**Table 1.** Key technological strategies for patient experience in EDs.

References	Dimension	Strategy category	Description	Measurement methods	Outcomes and impact	Challenges
Westphal et al [22]	Comm^a^ and ATI^b^	AI, Int & IS^c^	MyED: Web application providing real-time information about ED^d^ journeys, procedures and expected waiting times Target population: patients, family	Deployment survey, interviews, usage analytics	Improved patient understanding of the ED journey; positive reactions to comprehensive health information; enhanced transparency	Delays in synchronizing data from hospital databases; limited engagement period due to short ED visits
Marshall et al [16]	Comm	Int & IS	Digital whiteboards: E-paper displays showing real-time orienting and clinical information in ED rooms Target population: patients, Family	Satisfaction survey (Likert), custom quantitative perception survey	96% of patients preferred rooms with whiteboards; higher overall ED satisfaction; more likely to recommend hospital to family/friends	Not integrated with EHR^e^; updates not truly real-time; potential privacy concerns
Østervang et al [5]	Comm and ATI	Int & IS	Cetrea Clinical Logistics for patients: hospital information system displaying time estimates, staff names, and educational videos Target population: patients, family	SUS^f^ surveys (Likert), qualitative semistructured interviews	Increased sense of control reduced uncertainty; improved communication with health care professionals; positive impact on family members; usability rated close to excellent (83.6 SUS)	Difficult for older patients; privacy concerns; IT support dependency; content limitations
Steel et al [17]	ATI	Int & IS	MyEDCare: Mobile app for discharge instructions and information comprehension Target population: patients	ED Consumer Assessment of Health Care Providers and Systems patient satisfaction scores, usage analytics	Reduced 72-hour, 9-day, and 30-day unscheduled return ED visits; positive impact on patients’ perceptions of ED nursing; improved patient safety	Patient difficulties understanding complex information; technical issues; software bugs
Doyle et al [24]	Comm and ATI	Int & IS	PETS^g^/REDCap^h^ digital discharge system: discharge system: digital tools for pediatric patient registry and discharge information. Target population: patients, family	SUS scores (Likert), confidence surveys, semistructured interviews	Parents felt more confident with digital discharge information; preferred digital engagement for clinical research; high usability ratings (94 SUS)	Parent concerns about understanding instructions; technical issues
Jaafar et al [26]	Comm and ATI	I&H^i^	Patient Tracking System (TrackMe): Mobile app with wireless wristbands for real-time updates on patient location Target population: staff, family	Satisfaction surveys (Likert)	Enhanced family engagement; decreased anxiety through real-time updates about patient status and location; high user satisfaction (mean >4.0)	Resistance to adoption among staff; training needs; connectivity and infrastructure requirements

a Comm: communication.

b ATI: access to information.

c Int & IS: interfaces and information systems.

d ED: emergency department.

e EHR: electronic health record.

f SUS: System Usability Scale.

g PETS: Parent Engagement Through Technology Solutions.

h REDCap: Research Electronic Data Capture.

i I&H: infrastructure and hardware.

Common measurement methods include satisfaction surveys, usability scales, and structured interviews, reflecting a focus on patient-centered evaluation (see the *Measuring Usability, Patient Experience, and Satisfaction Improvement* subsection for more information). Reported outcomes and impact generally include improved understanding of care processes [22], reduced anxiety [5,26], and better communication with health care professionals [5], although challenges persist, such as technical limitations [17], integration with existing systems [16], and resistance to adoption [26]. Notably, infrastructure-based tools such as patient tracking systems [26] complement these efforts by supporting real-time updates, but they face barriers related to cost and implementation complexity. Overall, the evidence suggests a trend toward multidimensional strategies that prioritize transparency and communication while highlighting gaps in scalability and interoperability. In addition, the literature provides limited discussion on how these strategies can adapt to dynamic changes in ED protocols [22], ensure equity among diverse patient populations—including individuals with disabilities or lower digital literacy [5,18,24]—and effectively support digitally underserved or vulnerable groups [16,17].

#### Technologies and IT Infrastructures

Table 2 provides an overview of the main technologies and IT infrastructures used in ED settings, grouped by strategy category.

**Table 2.** Overview of technologies and IT infrastructures used in EDs, grouped by strategy category.

Strategy category and technology/infrastructure	Description and outcome	References
Interfaces and information systems		
Mobile app	Provides a patient-friendly health information and communication interface through a smartphone app, designed to deliver real-time and comprehensive medical data—such as health status, triage classification, vital signs, discharge instructions, and location within the ED^a^ journey—while also enabling communication with patients’ next of kin and supporting guidance for effective illness management, thereby improving health care services and enhancing patient satisfaction.	[17,20,26-28]
Web dashboard	Delivers a user-friendly, graphical web interface that provides real-time updates on patients’ medical status—including triage classification, clinical conditions, and vital signs—along with their ED treatment progression, open clinical note access, care pathways, and discharge instructions. This interface enhances transparency and patient engagement during the ED stay.	[5,14,18,19,22-25,28]
Digital whiteboard	Provides an electronic paper interface via a digital whiteboard that communicates relevant real-time clinical information to ED patients, including status updates, orientation details, care team assignments, safety alerts, patient orders, and disposition information. This interface enhances patient understanding of their journey through the ED.	[16]
Patient-centered information system	Provides an information system accessible across various devices and interfaces (web and desktop applications), designed to deliver real-time updates and detailed information—including procedures, treatments, associated waiting times, nurse and physician responsible for care, and current and upcoming stages—directly to patients and their family members. This enhances patients’ understanding of their individual journeys through the ED and supports informed decision-making and communication.	[5,22]
Infrastructure and hardware technologies		
Internet of Medical Things	Includes IoT^b^ devices (eg, wearables) for real-time monitoring of ED patients, providing vital insights into their health status, vital signs, patient stratification for special services, and physical location within the ED. This information supports clinical decision-making and enhances communication by delivering timely and essential updates to both medical staff and patients’ families about the health care process.	[26-28]
Computing infrastructure	Covers advanced computing infrastructures, such as cloud computing, that support real-time data storage, processing, and analysis in EDs.	[26,28]
Artificial intelligence–based		
ML^c^ algorithms	Encompasses ML algorithms like random forest for predictive analytics in EDs, providing insights into patient treatment, stratification, and predicting waiting times based on workload and patient characteristics.	[14,15,22]
Simulation-based		
Discrete-event simulation	Provides a simulation-based model in R to estimate individual treatment times from aggregated ED data, aiming to assess delays, improve waiting time estimates, and enhance understanding of patient flow.	[21]
Petri net model	Comprises a simulation-based model in Python using Colored Petri Nets to model and analyze ED processes and workflows, supporting improvements in patient flow across treatment paths and enhancing decision-making efficiency.	[15]

a ED: emergency department.

b IoT: Internet of Things.

c ML: machine learning.

*Interfaces and information systems* emerge as the most significant strategy category for directly improving patient experience through enhanced communication and transparency (14 studies). Within this category, *mobile* and *web dashboard applications* are the most widely adopted technologies (5 and 9 studies, respectively). For example, the Parent Engagement Through Technology Solutions (PETS) [24] and MyEDCare [17] mobile apps support patients and caregivers during discharge by improving comprehension and reinforcing key information about the ED journey. Complementing these mobile tools, the MyED system [22] and the Cetrea Clinical Logistics (CCL) dashboard for patients [5] were designed as web dashboard applications to enhance patients’ understanding of their care trajectory by displaying completed, ongoing, and anticipated procedures, as well as identifying the clinicians responsible for their care. Additionally, digital whiteboards, leveraging E-paper technology, deliver real-time notifications on delays and post-discharge instructions, functioning as integral components of the IT infrastructure [16].

Building on these interface-focused solutions, *infrastructure and hardware technologies* provide the foundation for real-time monitoring and data exchange. This category integrates *Internet of Medical Things* (*IoMT*) components with robust *computing infrastructure*. IoMT technologies enable real-time sensing and location tracking through dedicated hardware: beacon devices worn as patient bracelets and beacon readers (gateways) enable continuous wireless monitoring of patient location and status [26]; wearable biosensors capture physiological signals such as heart rate and oxygen saturation, supported by real-time location devices, Wi-Fi hotspots, and Bluetooth-to–Wi-Fi gateways for data transmission [28]; and near-field communication (NFC) tags paired with mobile electronic medical record (EMR) devices (eg, smartphones) allow location-based access to patient information [27]. Computing infrastructure complements these technologies by offering backend systems for secure data storage, processing, and decision support, including cloud-based databases and local servers operating within private networks [26,28]. Examples of strategies include TrackMe [26], which combines beacon-enabled bracelets with a mobile app and a 3-tier architecture for real-time updates; the NFC-integrated Mobile EMR System [27], which accelerates access to patient records; and IntelTriage [28], which uses wearable biosensors and LAN-based communication to monitor vital signs and geo-location, supported by a decision-support subsystem for dynamic prioritization.

Building on these physical infrastructures, *AI-based* strategies leverage computational techniques to enhance decision-making and operational efficiency. Predictive models such as *machine learning algorithms* [14,15] and random forest [22] approaches analyze EMR data—including length of stay, procedures, and clinical notes—to generate actionable insights. For example, the AI-enabled non-English language preference prioritization strategy [14] computes complexity scores for patients with a non-English language preference, while the AI/ML model presented in Gehlot et al [15] provides deeper insights into treatment details and patient experiences, thereby supporting more accurate patient routing based on acuity. Similarly, the MyED strategy [22] combines random forest models with operations research techniques, such as process discovery and queue mining, to predict waiting times for specific procedures and deliver real-time updates to patients through a responsive mobile interface.

Finally, *simulation-based* approaches complement predictive analytics by modeling complex ED workflows to optimize patient flow. Techniques such as *discrete-event simulation* and *Petri Net models* are used to estimate waiting times, identify bottlenecks, and validate algorithms for time-to-treatment predictions. These models support more reliable and equitable resource allocation. Reported strategies use these models to simulate care pathways and assess workflow constraints, enabling validation of predictive algorithms for waiting time and treatment prioritization [21]. A notable example is presented in Gehlot et al [15], where a Colored Petri Net–based simulation is integrated with Python-based AI/ML components to evaluate and refine triage processes, aiming to improve clinical outcomes and enhance patient experience.

Further details on usability, patient experience, and satisfaction impacts are provided in Table 1 and discussed in the subsection *Measuring Usability, Patient Experience, and Satisfaction Improvement*.

### Patient’s Preferred Information

The selected studies displayed a wide variety of topics that patients preferred on their journey through the ED. Specifically, patients preferred detailed information on their medical status and progress updates [18,26], including triage status [15,28], treatment, and discharge instructions [17,19,24]. Additionally, patients valued comprehensive medical information related to their vital signs [18,25,28], laboratory results [27] and medical orders [16,18], nursing care plans [15,25,28], and the nurse and physician responsible for care [5,16]. Patients also appreciated personalized medical information and updates on procedures, associated waiting times [5,21], as well as current and upcoming stages of their ED stay [16,22]. They also favored precise tracking of their location in the ED [26,27], safety, and disposition information [16]. These preferences highlight a significant trend toward personalized health care information, underscoring the need for technological strategies to focus on delivering precise, individualized health data and highlighting the importance of accuracy and clarity in the patient communication tools of the ED.

### Measuring Usability, Patient Experience, and Satisfaction Improvement

Usability evaluations are essential in health care technology to identify design flaws early and prevent ineffective implementations [24]. Among reviewed studies, the System Usability Scale (SUS) was the most frequently used instrument [5,18,24,27]. SUS is a 10-item Likert questionnaire that generates a score from 0 to 100, interpreted using a grading scheme (eg, >90: “A” [excellent]; <60: “F” [poor]). To illustrate, the PETS mobile discharge tool scored 94/100 (A grade—Excellent), indicating superior design and strong patient acceptance [24]. Similarly, the CCL patient information system achieved near-excellent usability with a mean SUS score of 83.6/100 (B+ grade—Acceptable) [5]. In contrast, NFC-integrated mobile EMR and REDCap eConsent interfaces scored 71.9/100 and 78/100 (B grade—acceptable), reflecting moderate usability and less direct impact on patient experience [24,27]. The electronic health record display for ED patient health status and care plan progress received a mean SUS score of 69.6 out of 100 (C grade—marginally acceptable) [18], suggesting limited effectiveness in improving patient experience.

While SUS provides a standardized measure, complementary approaches are often necessary to capture user perceptions more comprehensively. Likert-scale satisfaction surveys (typically 5- or 10-point) are commonly used to assess design quality, content relevance, and overall user-friendliness [16,25,26]. For example, the web application for emergency trauma patients, using a 10-point Likert scale, reported a mean usability score of 4.09 (SD 0.74) [25]. Similarly, the TrackMe patient-tracking system, using a 5-point Likert scale, reported a mean usability score of 4.0 (SD 1.2) and a mean satisfaction score of 4.4 (SD 1.0), indicating a generally positive user experience during ED waiting periods [26]. These findings reinforce that usability and satisfaction are interrelated but distinct constructs, underscoring the need for multidimensional assessment.

Patient experience and satisfaction are closely linked to usability, which strongly influences technology acceptance in clinical settings. In ED environments, these areas are typically assessed using mixed methods that combine SUS scores, Likert-scale measures, and validated instruments such as Hospital Consumer Assessment of Health Care Providers and Systems (CAHPS) and ED-CAHPS [16,17], complemented by semistructured interviews for contextual insights into patient perceptions [5,22,24]. For example, semistructured interviews in the MyED study showed that patients who used the solution demonstrated significantly better understanding of their ED journey compared with nonusers (*F*_8,299_=2.519; *P*=.01) [22]. Using satisfaction surveys, the digital whiteboards intervention improved patient-reported communication about delays (1.96 vs 2.48; *P*=.03) and clarity of post-discharge instructions (2.41 vs 2.83; *P*=.02), and users were more likely to recommend the facility (9.66 vs 9.22; *P*=.05) [16]. Operational benefits were also reported in the MyEDCare study: patients receiving digital discharge instructions had significantly fewer unscheduled ED returns at 72 hours and 30 days compared with those receiving standard paper-based discharge (*P*=.003 and *P<.*001) and higher ED-CAHPS scores in nursing communication domains (eg, “Nurses explain in a way you understand”: 86.5 vs 81.9; *P*=*.*03) [17].

Collectively, these findings underscore that usability is not merely a design metric but a critical driver of patient experience, satisfaction, and even clinical outcomes in ED technology implementations. In addition, the evidence shows that *Interfaces and Information Systems*—particularly *mobile and web applications*—provide the greatest added value for improving patient experience, primarily by enhancing communication, transparency, and comprehension.

## Discussion

### Principal Findings

The integration of advanced digital technologies in EDs represents a rapidly evolving but still unstructured field. Among the 16 studies included in this review, 9 reported interventions that achieved measurable improvements in communication, patient satisfaction, or reductions in service fragmentation [5,16-18,22,24-27]. Despite the promising results (see subsection *Measuring Usability, Patient Experience, and Satisfaction Improvement*), the absence of standardized frameworks, definitions, and evaluation models limits comparability and scalability across settings [29]. Developing a shared conceptual and operational framework is essential to ensure consistency in research and guide technology implementation within ED systems.

Patient-facing technologies such as mobile apps, real-time dashboards, and web-based platforms have enabled patients to access individualized health information, often in real time, enhancing their sense of involvement in emergency care [22]. Some tools demonstrated significant benefits—for example, MyEDCare improved patient comprehension and experience (86.5 vs 81.9; *P*=*.*03) [17], and digital whiteboards reduced perceived uncertainty (2.41 vs 2.83; *P*=*.*02) [16]. However, implementation challenges were common. The MyED system faced difficulties synchronizing live data from hospital databases, and short ED visits limited patient engagement [22]. Usability issues were also reported among older adults, who requested simplified functions [5]. These findings underscore the need for participatory design approaches that incorporate patient perspectives prior to implementation [30]. Ultimately, usability remains a critical determinant of successful adoption and effective use [31].

Technological impact extends beyond patient interaction to clinical workflows. Real-time data systems and mobile-based discharge tools require adjustments in routines, new training protocols, and additional coordination. While these tools can enhance communication and data accuracy, they often introduce extra responsibilities, increasing cognitive and operational burden on health care professionals [24]. This tension—between intended benefits such as efficiency and the reality of added workload—has been documented in multiple studies [32,33]. Understanding nursing staff perceptions and acceptance is therefore essential for successful adoption [34]. Policymakers and ED managers must consider these trade-offs when planning technological strategies to avoid overburdening staff while pursuing efficiency goals [16].

Additionally, the integration of wearable devices (eg, TrackMe, IntelTriage) and patient-facing platforms (eg, CCL, MyED) is reshaping care dynamics by fostering patient autonomy [5] and involvement in decision-making [20,23]. Although these innovations hold promise for improved outcomes and satisfaction [16,17,23], they raise concerns regarding workflow implications (eg, TrackMe increased nurses’ workload [26]; fears of delays with MyED [22]), digital literacy barriers (eg, limited access to Open Notes among non-English speakers [23]; usability issues for older users of CCL [5]), and communication norms (eg, risks of patients viewing information before staff [22]; potential reduction in nurse/physician time) [5]). These factors must be addressed to ensure equitable and safe implementation.

Finally, the economic cost required for the investment, implementation, and maintenance of advanced technologies in EDs must be carefully considered. These expenses may include infrastructure upgrades, software development, technical support, and training for health care personnel [19]. Given the potential financial implications, future studies should explore the cost-benefit relationship of these technological strategies, assessing their feasibility, sustainability, and overall value in diverse health care settings [17].

### Strengths and Limitations

A key strength of this study is its multidimensional approach, which provides a holistic view by examining various facets of ED care, including communication and information accessibility. This comprehensive perspective was achieved by focusing on emerging technologies, such as AI and IoT, which represent the forefront of operational efficiency and diagnostic accuracy in EDs. The recent literature (2018‐2024) grounds the study’s findings in current technological trends, ensuring relevance. However, limitations include a focus on English articles, which may have excluded relevant studies in other languages, potentially restricting generalization. Furthermore, since many technologies analyzed are relatively new (eg, IoMT, AI), few studies directly address their long-term impacts. Variability in environments where these technologies are implemented can also affect reproducibility, especially in resource-limited settings. The recent temporal range may not capture emerging trends or long-term effects, highlighting the need for ongoing research to understand their sustained impact.

### Conclusions

This scoping review synthesized current technological strategies designed to enhance patient experiences in EDs, primarily through improved communication and access to information. The limited number of studies identified (n=16, published between 2018 and 2024) underscores the nascent and fragmented nature of this research area. Existing evidence suggests that patient-facing tools—such as mobile apps, real-time dashboards, and web-based platforms—can improve information transparency and engagement, while clinical workflow solutions, including digital whiteboards and mobile discharge systems, support communication accuracy and care coordination. Emerging innovations like IoT-wearable monitoring devices further demonstrate potential for promoting patient autonomy and continuous observation. However, these benefits are tempered by persistent implementation challenges. Usability barriers among older adults, technical integration issues, and increased workload for health care professionals remain significant obstacles. Equity concerns related to digital literacy and language accessibility further complicate adoption, raising questions about inclusiveness and fairness in technology-driven care. Importantly, none of the reviewed studies conducted systematic evaluations of long-term outcomes or cost-effectiveness across diverse ED contexts, limiting the evidence base for sustainable integration. Future research should move beyond descriptive assessments toward rigorous, standardized evaluation frameworks that capture clinical, economic, and experiential outcomes. Participatory and inclusive design approaches are essential to address usability and equity gaps, while comprehensive cost-effectiveness analyses will inform scalability and resource allocation. Advancing this agenda is critical to ensure that technological innovations in EDs are not only effective but also equitable, sustainable, and adaptable across varied health care settings.

## Supplementary material

10.2196/79782 Multimedia Appendix 1 Database search queries and results.

10.2196/79782 Multimedia Appendix 2 Crowe Critical Appraisal results.

10.2196/79782 Checklist 1 PRISMA-ScR checklist.

## Abbreviations

AI: artificial intelligence
CAHPS: Consumer Assessment of Health Care Providers and Systems
CCAT: Crowe Critical Appraisal Tool
CCL: Cetrea Clinical Logistics
ED: emergency department
EMR: electronic medical record
IoMT: Internet of Medical Things
ML: machine learning
NFC: near-field communication
PETS: Parent Engagement through Technology Solutions
PRISMA-ScR: Preferred Reporting Items for Systematic Reviews and Meta-Analyses Extension for Scoping Reviews
RQ: research question
SUS: System Usability Scale
