# Supplementary material for: Technological Strategies for the Patient Experience in Emergency Departments: Scoping Review
Source: JMIR Med Inform. 2026 Mar 9;14:e79782. doi: 10.2196/79782 (PMC12978909; doi:10.2196/79782)
Supplement: Multimedia Appendix 1 [file medinform-v14-e79782-s001.docx]

**Multimedia Appendix 1**

**Table 1.** Search equations and results across various databases.

| Database | Search Equation | Results (2018-2024) |
| --- | --- | --- |
| Scopus | TITLE-ABS-KEY (("Technolog*") AND ("Strateg*" OR "Approach*" OR "Solution*" OR "Framework*" OR "System*" OR "Model*") AND ("Emergency Department*" OR "Emergency Service, Hospital" OR "Emergency Room*" OR "Emergency Medical Service*") AND ("Communication" OR "Access to Information")) | 348 |
| PubMed | (("Technolog*") AND ("Strateg*" OR "Approach*" OR "Solution*" OR "Framework*" OR "System*" OR "Model*") AND ("Emergency Department*" OR "Emergency Service, Hospital" OR "Emergency Room*" OR "Emergency Medical Service*") AND ("Communication" OR "Access to Information")) | 394 |
| IEEE Xplore | (("Technolog*") AND ("Strateg*" OR "Approach*" OR "Solution*" OR "Framework*" OR "System*" OR "Model*") AND ("Emergency Department*" OR "Emergency Service, Hospital" OR "Emergency Room*" OR "Emergency Medical Service*") AND ("Communication" OR "Access to Information")) | 115 |
| CINAHL | (("Technolog*") AND ("Strateg*" OR "Approach*" OR "Solution*" OR "Framework*" OR "System*" OR "Model*") AND ("Emergency Department*" OR "Emergency Service, Hospital" OR "Emergency Room*" OR "Emergency Medical Service*") AND ("Communication" OR "Access to Information")) | 119 |
